# Retraction: 2, 3, 7, 8-Tetrachlorodibenzo-P-Dioxin (TCDD) Induces Premature Senescence in Human and Rodent Neuronal Cells via ROS-Dependent Mechanisms

**DOI:** 10.1371/journal.pone.0345512

**Published:** 2026-03-23

**Authors:** The PLOS One Editors

Following the publication of this article [1] concerns were raised with the results presented in Figs 1-3, and 8. Specifically,

- The following panels appear to partially overlap:
  - Fig 1A 72h 1nM and 50nM 0h.
  - Fig 1A 72h 50nM, 72h 100nM when rotated, and 50nM 72h when rotated.
  - Fig 8A 10nM TCDD and 30nM TCDD.
- In the Fig 2C p-Rb blot, lanes 1 and 2 appear more similar than would be expected from independent results.
- Similarities were noted between multiple background regions within the following blots:
  - Fig 3A p-Rb.
  - Fig 3A GAPDH.
- There appear to be irregularities in the background of the Fig 8B p-RB panel.

First author CW stated that they were responding on behalf of all co-authors. They stated that the panel overlaps in Figs 1A and 8A were the result of errors made during figure preparation as the images were sequentially numbered without clear group identifiers. They provided triplicate underlying image data from the time of the original experiments for all Fig 1A results and the Fig 8A 30nM results. The explanation of the data labeling procedure raises concerns about the reliability of the overall data handling practices, and in the absence of the full underlying data for Fig 8A, the reliability of the associated quantified results cannot be verified. In addition, editorial assessment of the underlying image data provided for Fig 1A raised additional concerns about the reliability of the published results.

First author CW stated that the original blots underlying Figs 2C and 3A are no longer available. In the absence of these underlying blots, the concerns with Figs 2C and 3A cannot be resolved and the reliability of the associated quantified results presented in Figs 2D and 3B cannot be verified.

Regarding the irregularity in the background of the Fig 8B p-Rb panel, first author CW stated that a background layer was inadvertently added during the final assembly of the published figure. Although the original blots for Fig 8B are no longer available, an earlier version of the figure was provided which supports the published results. PLOS considers the concern with Fig 8B resolved.

The *PLOS One* Editors retract this article in light of the above concerns with Figs 1A, 2C, 3A, and 8A, which call into question the reliability and integrity of the published results.

CW agreed with the retraction. JL, XN, JZ, SZ, ZD, CT, LL, and GX either did not respond directly or could not be reached.
